# Prevalence and associated factors of urinary incontinence in women living in China: a literature review

**DOI:** 10.1186/s12894-020-00735-x

**Published:** 2020-10-14

**Authors:** Kaikai Xue, Mary H. Palmer, Fang Zhou

**Affiliations:** 1 School of Nursing, Xuzhou Medical University, Xuzhou, China; 2 School of Nursing and Midwifery, Jiangsu College of Nursing, Huai’an, China; 3 School of Nursing, University of North Carolina at Chapel Hill, CB 7460, Chapel Hill, USA

**Keywords:** Women, Urinary incontinence, Bladder, Prevalence, China

## Abstract

**Background:**

This review of studies on urinary incontinence (UI) was focused primarily on UI prevalence rates and associated factors across the adult lifecourse of Chinese women. UI is a urologic symptom that can have a significant impact on women's physical and mental health and quality of life. In addition, women with UI may experience socioeconomic burdens due to UI’s effect on their ability to work and function in society. Although researchers from many countries have reported prevalence rates and associated factors for UI, little is known about the prevalence of UI in China’s large female population. Language may act as a barrier to the inclusion of published studies in English-language journals. To overcome this barrier and to add to the global knowledge base about UI in women, the authors reviewed and discussed findings from epidemiological studies published in China and in Chinese language.

**Methods:**

The authors retrieved research studies from the Wanfang database using the following search terms: "Subject: (Female) × Subject: (Urinary incontinence) × Subject: (Prevalence) × Date: 2013 to 2019". Searches employed the China National Knowledge Infrastructure Database, VIP Database for Chinese Technical Periodicals and China Biology Medicine Database. The authors also used PubMed to search English-language studies published in Chinese journals on UI in Chinese women.

**Results:**

This literature review includes 48 articles published between January 2013 and December 2019. The overall UI prevalence rates reported in adult Chinese women ranged from 8.7 to 69.8%, representing 43–349 million women, respectively. For women aged 17–40 years, 41–59 years, and 60 years and older, prevalence rates ranged from 2.6–30.0, 8.7–47.7, to 16.9–61.6%, respectively. Significant associated factors for overall UI included age, body mass index, constipation, parity, and menopause. Despite the 17–40 age range being peak reproductive years, the literature revealed little focus on UI prevalence rates. For women aged 41–59 years, the main associated factors included those related to pregnancy and gynecologic diseases. For women 60 years and older, chronic diseases represented most of the associated factors.

**Conclusions:**

About 43–349 million Chinese women may experience UI. Many of the identified associated factors could be mitigated to reduce UI incidence and prevalence rates. Little is known about the prevalence rates and associated factors for UI among young (aged 17–40) Chinese women. Future research should investigate UI in young women to improve bladder health across their lifecourse.

## Background

Urinary incontinence (UI), which is defined as the complaint of the involuntary loss of urine [1], is one of the most frequently reported lower urinary tract symptoms in women [2]. The three main types of UI are stress urinary incontinence (SUI), urgency urinary incontinence (UUI), and mixed urinary incontinence (MUI) [1]. The definition of each UI type is as follows: SUI is “the complaint of involuntary loss of urine on effort or physical exertion (e.g., sporting activities) or on sneezing or coughing”; UUI is the “observation of involuntary leakage from the urethra synchronous with the sensation of a sudden, compelling desire to void that is difficult to defer”; and MUI is the “complaint of involuntary loss of urine associated with urgency and also with effort or physical exertion or on sneezing or coughing” [1].

Prevalence rates of UI for women reported globally can differ as a result of variations in methods used in studies or reports, women’s underreporting of their symptoms, and providers underdiagnosing the condition [3]. As an example, the UI prevalence rate for women between 45 and 60 years old living in Brazil was 23.6% [4], whereas the prevalence rates for adult women (over 18 years old) in Germany, Denmark, and Norway were 48.3%, 46.4% [5], and 18.7% [6], respectively. The number of women with UI in the United States has been estimated to be about 28.4 million [7]. Previous research suggests that 31.9% (approximately 160 million) women in China are affected by UI [8], indicating an urgent need for health and social resources to manage and treat UI.

Many factors are associated with UI [8], including unmodifiable factors (e.g., age, gender, menopause, history of vaginal delivery) and potentially modifiable factors (e.g., smoking, alcohol intake, toileting behaviors [9], constipation, and obesity). Besides UI’s impact on women’s physical and mental health, UI affects women’s quality of life by limiting social activities [10] and interactions, interfering with the ability to work [11], and increasing the financial burden on women and society [12–14]. Therefore, UI should be viewed as both a women’s health issue and a public health issue [15].

Studies of UI prevalence rates and associated factors often appear in English-language journals, but research findings published in non-English-language journals or English-language journals not published outside of China are seldom disseminated widely. The resultant knowledge gap could negatively affect potential research and clinical advances with regard to Chinese women’s bladder health. This gap could also delay the development of culturally appropriate interventions to prevent and treat UI across women’s lifecourse. Thus, the need to close the knowledge gap is important, especially considering China’s large female population. For example, 650 million women were living in China in 2010, with more than 500 million women over 20 years old [16].

The aims of this study were to: (1) summarize findings from studies in non-English-language journals and English-language journals published in China that investigate UI prevalence in Chinese women, (2) categorize the findings by life stage (i.e., age range categories), and (3) facilitate dissemination of this existing information to researchers and clinicians to aid in their planning to prevent, manage, and treat female UI.

## Methods

### Literature search

The authors searched the relevant literature using five databases: the Wanfang full-text database (a Chinese professional academic database covering journals, meeting minutes, papers, academic achievements and academic conference papers), China National Knowledge Infrastructure (CNKI) Database (the largest continuously updated China journal full-text database in the world. It contains more than 9100 important journals in China, mainly including academic, technical, policy guidance, higher science popularization and education, and some basic education, popular science and technology, popular culture and literature and art works, covering natural science, engineering technology, agriculture, philosophy, medicine, humanities and Social Sciences and other fields, there are more than 32.52 million full-text documents), VIP Database (it analyzes the contents and citations of more than 14,000 kinds of science and technology periodicals and 57 million full-text periodicals published in China), and China Biology Medicine database (it involves basic medicine, clinical medicine, preventive medicine, pharmacy, traditional Chinese medicine, traditional Chinese medicine and other biomedical fields. It is an important retrieval tool for medical literature in China at present) for Chinese Technical Periodicals, and PubMed for English-language articles. The Wanfang full-text database search expression was Subject (Female/Women) × Subject (Urinary incontinence) × Subject (Prevalence) × Date: 2013–2019 or Subject: (Lower urinary tract symptoms) × Subject (Prevalence) × Date: 2013 to2019. The authors used the same search strategy for the other four databases: China National Knowledge Infrastructure, VIP, China Biology Medicine, and PubMed. After the electronic retrieval of relevant articles, the authors obtained further studies from the references cited in those articles. The search was conducted in two phases: initially the review included studies published between January 2013 and December 2017, and was subsequently updated to include literature published between January 2018 and December 2019.

### Eligibility criteria

The inclusion criteria for this review were: (1) studies must be a cross-sectional research design; (2) study participants were adult women (≥ 17 years old) living in China; (3) studies discussed prevalence rates and/or associated factors of UI; and (4) sample sizes were greater than or equal to 100 women. The exclusion criteria were (1) narrative or systematic reviews, meta-analyses, or clinical guidelines; (2) case–control studies of UI treatment or care; and (3) studies focused on UI mechanisms. (4) Study participants were currently pregnant or up to 3 months postpartum.

### Study selection

**Fig. 1:**
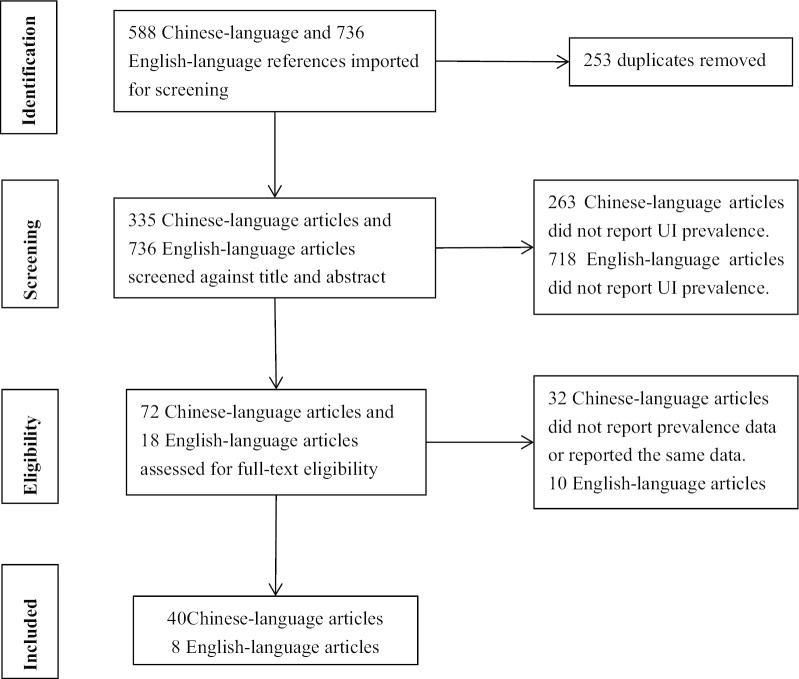
PRISMA flow diagram for the review process

Two native Chinese-speaking reviewers (proficient in English) independently screened the article titles and abstracts. Duplicate articles were excluded. Full texts were obtained for the selected studies to assess their eligibility and their reference lists were scanned for further relevant articles. Any disagreement that arose between the reviewers regarding the inclusion or exclusion of articles was resolved through discussion. See Fig. 1.

### Data extraction and analysis

The study team developed a standardized abstraction table. Data extraction was performed by two reviewers. One author independently read the included studies and extracted data from them, but consulted with the other authors to resolve ambiguities. The studies were described and then summarized using a narrative descriptive approach. Data in the abstraction table were double checked by reviewers.

The age ranges of the participants differed among the studies selected for review. Thus, we adopted the following age range categories to examine the studies more closely in terms of participant age: 17–40 years old for young women, 41–59 years old for middle-aged women, and 60 years old and over for older women. Two reviewers independently extracted data onto a data extraction summary sheet regarding prevalence rates and associated factors for UI in young, middle-aged, and older Chinese women. Factors that were significantly associated with urinary incontinence (*p* < 0.05) were included in the review.

## Results

The articles initially retrieved included 335 Chinese-language and 736 English-language articles, after removing 253 duplicates. Of those articles, 72 Chinese-language articles and 18 English-language articles were retained after screening titles and abstracts. We then reviewed the full texts of each article and identified 40 Chinese-language and 8 English-language articles for final analyses (Fig. 1). Among the Chinese-language articles, there were four Master's thesis [17–20].

**Fig. 2:**
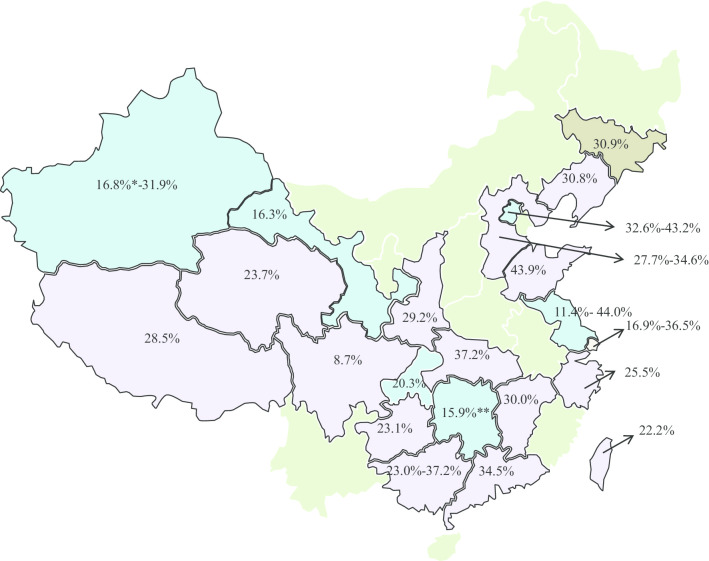
Prevalence rates of female urinary incontinence by location in China (This figure is created by authors with Word of Microsoft Office Home and Student 2019 (https://www.microsoftstore.com.cn/office/office-home-student-2019). It does not represent all the territory of China (China Sea Islands etc.).).  UI (UI: Urinary incontinence, type not specified) of adult women (≥ 18 or 20 years old).  SUI (SUI: Stress urinary incontinence) of adult women (≥ 18 years old).  UI of older women (≥ 60 or older).  No prevalence of UI reported. Two studies including women with 17 years old: *[20]. **[40]

Studies selected for review were conducted in 22 different provinces and regions in China including: Shanghai [21], Beijing [22], Chongqing [23], Hebei [24], Shanxi [25], Gansu [26], Xinjiang [27, 28], Guangzhou [29], and Taiwan [30, 31] etc. Figure 2 presents a map of China that reported prevalence rates of UI in various areas throughout the country.

Based on the eligibility criteria, all the included articles were cross-sectional research design. The sample sizes range from 146 to 18,992. Almost all of the studies reported that trained interviewers surveyed face to face with standardized questionnaire and some of the researchers also conducted gynecological examination for participants [32–34]. All the studies focused on the associated factors of any UI or SUI. Two article focused on the associated factors of the other type (UUI) [19, 22]. No articles that focused on the associated factors of MUI were located.

The prevalence rates and associated factors for female UI in China are presented in the “Appendix”. The reported prevalence rates of overall UI (overall UI includes all types of UI) in Chinese adult women ranged from 8.7 [34] to 69.8% [35]. Where prevalence of specific UI types were reported, the following ranges were: SUI prevalence rates ranged from 6.7 [34] to 44.0% [35], UUI prevalence rates ranged from 1.2 [17] to 21.0% [22], and MUI prevalence rates ranged from 1.5 [17] to 15.7% [36]).

Definitions for UI, SUI, UUI, and MUI differed across some of the studies. Most of the authors used the International Continence Society (ICS) definition of UI: “complaint of involuntary loss of urine” [1]. Five studies [37–41] did not include a definition for UI. Other definitions used varied slightly from the ICS definition, including: (1) UUI was defined as, “the occurrence of urinary frequency, urgency, increased frequency of nocturnal discharge and decreased urine output; or cannot control urine leaking out, waited too late to urinate leading to leakage of urine” [42, 43]; and (2) UUI was defined as, “urinating without any warning or a weak or faint amount of early warning, sudden urge sensation resulting in uncontrolled urine outflow” [22]. SUI was defined as “urine leaks out when exercising” [22]. The “Appendix” includes the UI definitions used in the reviewed studies.

Only one article reported UI prevalence rates and associated factors specifically for young women (18–26 years old) [44]. It found that age (21–26 years old comparing with 18–20 years old) (OR = 0.87, 95% CI = 0.77–0.98), constipation (OR = 2.40, 95% CI = 1.49–3.84), alcohol consumption (OR = 1.76, 95% CI = 1.11–2.79), often/always delayed urination (OR = 1.74, 95% CI = 1.31–2.31), and/or often/always strained to urinate (OR = 1.43, 95% CI = 1.11–1.85) were associated with UI. The prevalence of UI in this study was 23.6% [44]. Based on the data extracted from studies that included adult women, UI prevalence rates for community-living young women (18 to40 years old) ranged from 2.6 [45] to 30.0% [46], and the prevalence rates of SUI, UUI, and MUI were reported as 4.7 [21]–24.5% [47], 0 [48]–8.1% [42], and 0.7 [49]–10.7% [50], respectively. Eight articles [29, 30, 34, 51–55] were focused on middle-aged, perimenopausal, and menopausal women, and seven articles [41, 43, 56–60] were focused on older women. The prevalence rates of UI for middle-aged women ranged from 8.7 [34] to 47.7% [36], and the prevalence rates of UI for older women ranged from 16.9 [57] to 61.6% [60]. Table 1 provides a summary of the UI prevalence rates and associated factors of different age group of women. Table 2 reports the number of articles that investigated underlying factors and number of articles in which these factors were found to be significantly associated.

**Table 1:** Prevalence Rates and Factors Associated with Urinary Incontinence in Chinese Women

Population	Age	Prevalence	Associated factors
Young women	17^#^ to 40 years	UI 2.6–30.0%	Age, constipation, alcohol consumption, delayed urination, strained to urinate
		SUI 4.7–24.5%	
		UUI 0–8.1%	
		MUI 0.7–10.7%	
Middle-aged women	41–59 years	UI 8.7–47.7%	Age, overweight, BMI, level of education, monthly income, constipation, menstrual disorders, menstrual disorders, parity, perineal laceration, postpartum urinary incontinence, pelvic operation history, POP, menopause, atrophic vaginitis, history of hormone replacement therapy, urinary tract infection, mental disease, hyperlipidemia, chronic bronchitis or asthma, respiratory system diseases, fecal incontinence
		SUI 6.7–40.5%	
		UUI 2.1–62.9%	
		MUI 1.2–20.4%	
Older women	≥ 60 years	UI 16.9–61.6%	Age, more than 80 years old, BMI, low level of education, spouse, mental labor, alcohol consumption, delayed voiding, medical payment method, constipation, parity, pregnancy > 4 times, leakage of urine, gynecological diseases, urinary tract infection, malnutrition, autonomic activity limitation, activities of daily living, chronic pain, sleep disorders, chronic respiratory, urinary, cardiovascular disease, diabetes, drugs, indwelling catheterization
		SUI 10.0–62.4%	
		UUI 3.3–21.1%	
		MUI 3.3–26.6%	

*UI* urinary incontinence, *SUI* stress urinary incontinence, *UUI* urgency urinary incontinence, *MUI* mixed urinary incontinence, *BMI* Body Mass Index, *POP* pelvic organ prolapse

**Table 2:** Number of articles investigated underlying factors and number of articles in which the factors significantly associated with urinary incontinence (N = 48)

Factors^a^	Number of articles^b^	Significantly associated^c^
Age	37	29
BMI/weight	28	22
Constipation	27	19
Parity	23	15
Vaginal delivery	23	10
Hypertension	22	8
Menopause	21	8
Educational	19	8
POP	15	8
Pelvic surgery/hysterectomy	20	7
Alcohol consumption	17	7
Chronic pelvic pain	8	7
Urinary tract infections	8	7
Diabetes	23	6
Cough	8	6
Smoking	18	5
Occupation	13	5

*POP* pelvic organ prolapse

^a^Factors: the top seventeen associated factors according to the investigated frequencies

^b^Number of articles: the number of articles that investigated underlying factors

^c^Significantly associated: number of articles in which these factors were found to be significantly associated with urinary incontinence

## Discussion

The studies selected for this review revealed a wide range of prevalence rates for overall UI and the specific types of UI, which may be due, in part, to the country’s size. China is a large country with 9.6 million square kilometers and it includes numerous cultures in urban and rural locations. Genetic factors, diet, lifestyle, local environment, climate, economic development level, occupation types, and toileting behaviors may differ across these regions. These factors could act as determinants of UI, and thus affect variation in UI prevalence rates. Other important reasons for the variations in UI prevalence rates and UI types are the different research definitions and statistical and sampling methods used in the selected studies. For example, researchers used different methods when creating samples (i.e., based on general outpatient [40] or gynecological clinics [38] or physical examination [61] or nursing institutions [60]). The study participants had different occupations (i.e., medical personnel [62], nurses [22] and railway workers [63]), and different living conditions (i.e., rural [64] and urban [45]). Also, in some studies, clinicians conducted physical examinations and documented medical histories, which may have yielded different results from women’s self-reported questionnaires.

Researchers identified several factors associated with overall UI in Chinese women. Some of these factors are modifiable, such as weight, BMI, education, smoking and drinking alcohol. Some factors can be remedied or controlled (i.e., constipation, hypertension, chronic cough, diabetes, respiratory diseases, and vaginitis). Lastly, although some of the identified factors are unmodifiable (i.e., age, vaginal delivery, cesarean section delivery, and menopause), interventions can nonetheless be designed and tested to promote bladder health and help delay the onset or slow worsening of UI.

Age is often associated with UI risk factors such as education level, number of pregnancies and deliveries, menopause, and chronic conditions such as hypertension, diabetes, and respiratory diseases. Thus, studying clusters of factors that increase UI risk across the lifecourse is important. Moreover, using a lifecourse perspective is advocated in bladder health research [65].

Chinese scholars have largely confined their research on prevalence of UI in adult women (≥ 17 years old), but in their findings of subgroup analyses for discrete age groups were not reported. Despite this limitation, when using the prevalence rate range of 2.6 [45]–30% [46] for young women aged 17–40 years old, the number of Chinese women in this age group who are affected by UI is estimated to be between 6.6 million and 75.8 million. This finding alone indicates that screening young women for risk factors, especially modifiable ones, and taking actions to minimize or eliminate the effects of these factors could potentially prevent or delay incident cases of UI throughout the lifecourse and especially later in life.

In a prospective cluster-randomized controlled trial of UI among young women (18–40 years), SUI prevalence was 14.3% [66]; 49.5% of these women had SUI during pregnancy, 43.6% had postpartum SUI, and 6.9% had SUI before pregnancy. Because muscle, connective, and nervous system pelvic structures are subjected to anatomical, morphological, functional, and hormonal changes during pregnancy, clinicians should initiate primary prevention interventions [67]. The pelvic floor also undergoes an enormous amount of stretching to allow the passage of a newborn during vaginal delivery [68]. Evident or hidden injuries to the pelvic floor may manifest as urinary and fecal incontinence, prolapse symptoms, or sexual dysfunction, all of which have a considerable impact on quality of life. Because pregnancy and childbirth can put young women at risk of developing UI [69], research and clinical attention should be focused on understanding the underlying mechanisms of UI as well as developing effective strategies (perform pelvic floor muscle training, maintain normal weight, avoid constipation [70]) to preserve bladder health for young Chinese nulligravid women.

In our research, there was only one study focused on the young women (18–26 years old) and it found that age (21–26 years old comparing with 18–20 years old), constipation, alcohol consumption, often/always delayed urination, and/or often/always strained to urinate were associated with UI. The prevalence of UI in this study was 23.6% [44]. Evidence from studies of young women who live outside of China also provides compelling evidence that UI in young women should be investigated further and intentionally. For example, the prevalence rate of UI for Swedish women (*N* = 653) between the ages of 18 and 30 years was 12% [71]. In nulligravid Australian women aged 16–30 years (average age 22.5 ± 3.2 years), the prevalence of overall UI was 12.6% [72], and women who were sexually active and those who were not using oral contraceptives had the highest rates of UI [72]. Mishra et al.’s study found that the UI prevalence rate for Australian women aged 22–27 years was 6.8% at baseline and increased to 16.5% nine years later [73]. These researchers also reported that women with depressive symptoms or a history of depression were more likely than those without depressive symptoms to report subsequent UI symptoms [73]. For nulligravid women living in Italy between 15 and 25 years old, age, BMI, depression/anxiety/panic attacks, eating disorders, and constipation were risk factors for UI [74]. Participating in organized sports that involves high-volume exercise for competition also increased the risk of developing UI (OR = 2.53, 95% CI = 1.3–2.7) [75]. Other studies conducted outside of China showed that UI is an issue for many nulliparous female athletes [76].

Cultural differences may be evident with regard to UI risk and associated factors in China and abroad. A relatively new factor under investigation is toileting behaviors, i.e., actions women take immediately prior to and during urination [77–80]. Toileting behaviors play a role in developing or worsening urinary symptoms, but more research is required, especially studies that focus on young women in China [44]. It is often during youth and young adulthood when women develop habitual behaviors and form beliefs and attitudes about bladder health for themselves and their children. This period in women’s lifecourse may be pivotal in influencing prevalence rates because evidence is mounting that research to prevent or reduce UI in this age stage is important [65].

This review also found that UI prevalence rates for middle-aged Chinese women ranged from 8.7 [34] to 47.7% [36], which represents 15.5–85.0 million middle-aged women with UI. The UI prevalence rate for women between 45 and 60 years old living in Brazil was 23.6% [4]. The associated factors relate mainly to obstetrics-related ones, such as parity, perineal laceration, and postpartum UI, and gynecological factors, such as menstrual disorder, menopause, pelvic organ prolapse, pelvic operation history, and hormone replacement therapy (see Table 1). UI prevalence rate was found to be significantly higher in a postmenopausal group than a premenopausal age group [81], which may be related to the change of hormone levels in postmenopausal women [82]. Research conducted in China found that the protective effect of cesarean section delivery compared with vaginal delivery was more obvious at five years postpartum than at one year postpartum [83]. Vaginal delivery appears to aggravate pelvic floor structure injuries. Compared to this study, studies conducted in other countries had similar findings. In Norway, a survey of middle-aged women (average age 47 years) who had delivered either vaginally or by Caesarian sections 15–23 years previously had 46.9% UI prevalence. In addition, caesarian section delivery was a protective factor as UI prevalence was lower than in this group of women as compared to women who delivered vaginally.[84]. Further research is needed to determine the mechanism(s) of injury during childbirth and identify associated factors and interventions that prevent or lessen adverse effects of childbirth on bladder health.

Although UI is common across the lifecourse, its prevalence peaks in the older age group of women [3]. China's older female UI prevalence rate ranged from 16.9 [57] to 61.6% [60], which translates to more than 12.8–46.7 million older Chinese women living with UI. Scholars outside of China have found similar UI prevalence rates for older women. A survey of 622 older women living in Brazil (average age 64 years) revealed that the UI prevalence rate was 52.3% [85] and for women over 65 years old living in Turkey the UI prevalence was 51.6% [86]. In China, risk factors associated with UI include being 80 years old and over, BMI, malnutrition, low educational level, sleep disorders [58], unspecified number and types of medications, history of pregnancy, urine leakage during pregnancy, chronic respiratory disease, cardiovascular disease, gynecological diseases, diabetes, urinary tract diseases [31], chronic pain, constipation, and restricted activity [41] (see Table1). Compared to middle-aged women, older women have a higher prevalence of chronic diseases (i.e., respiratory disease, hypertension, diabetes, etc.), limited physical activity, malnutrition, and other factors that could be contributing risk factors for UI. A longitudinal study of older women (baseline ages of 51–74 years) conducted in the United States years who did not have UI found the incidence of UI was 37.2% ten years later [87]. This study also found that UI prevalence in later in life had strong associations with obesity, functional ability, and medical comorbidities, but not with parity [87].

Although we used rigorous methods to conduct this epidemiological review, some limitations are noted. First, although we searched one English database (i.e., PubMed), and the PRISMA review process was followed, we may have inadvertently overlooked eligible articles. Second, research into UI prevalence rates has not been conducted in all 34 provinces in China (e.g., 22 of the 34 provinces are represented in the included studies), which could result in an incomplete picture of the distribution of UI across the Chinese female adult population. The quality of the included studies was not formally assessed in order to include a broad representation of the literature. It is recommended that future studies include this important element. This paper, however, provides important information and raises awareness about prevalence of UI in women living in China.

## Conclusions

Most of the information in this review has been previously unavailable to researchers in countries outside of China. Thus, in addition to adding to the specific knowledge about UI in Chinese women, this review adds to the global knowledge base about female UI. Findings from the reviewed studies revealed that UI prevalence rates for Chinese women range from 8.7 to 69.8%. Most of the studies focused on middle-aged and older women. Little information about UI prevalence for women in their teens and twenties was found. Given the prevalence of UI and size of China’s female population, research is needed to better understand the risk, and protective, factors for UI across the lifecourse of women. This information should stimulate interest in further multi-country comparisons and the development and testing of evidence-based interventions to prevent UI.

## Data Availability

All data generated or analyzed during this study are included in this published article.

## Appendix

See Table 3.

**Table 3:** Prevalence and associated factors of urinary incontinence for three age groups

First author	Date range of study	Location (provinces)	Age (characteristic)	Instrument (methods)^a^	Prevalence (n/N (%))	Authors’ definitions of UI and UI types	Associated factors [odds ratio (OR), 95% confidence interval (95% CI)]
Zhang and Lei [8]	February 2006–July 2006	Northwest, Northeast, North, Southwest, East, and South-central China	$>$ 20 years	ICIQ-FLUTS^b^ (face to face interviews by trained interviewers using a standardized questionnaire)	UI 6052/18992 (31.9)	ICS^c^ definitions	UI: Age 30–39 years (1.55, 1.34–1.80), 40–49 years (2.66, 2.29–3.10), 50–59 years (2.80, 2.32–3.38), 60–69 years (2.46, 1.98–3.06), 70–79 years (3.02, 2.39–3.81), 80–89 years (3.32, 2.52–4.31), ≥ 90 years (5.87, 3.58–9.65), BMI ≥ 28 kg/m^2^ (1.77, 1.52–2.06), menopause (1.41, 1.24–1.61), residence (urban: 0.90, 0.84–0.97), constipation (1.87, 1.70–2.06), pelvic organ prolapse (POP) (1.96, 1.47–2.60), hypertension (1.29, 1.16–1.44), alcoholism (1.51, 1.35–1.68), vaginal delivery (1.63, 1.49–1.79)
					SUI 3592/18992 (18.9)		
					UUI 488/18992 (2.6)		
					MUI 1788/18992 (9.4)		
Sun and Wenyu [17]	2012–2013	Qinghai	20–80 years	(Face to face) Bristol Standardized Questionnaire-FLUTS)	UI 253/1066 (23.7)	ICS definitions	UI: Age (1.08, 1.05–1.12), natural vaginal delivery (0.002, 0.00–0.01), obstetric forceps vagina delivery (8.17, 1.49–44.77), parity (668.8, 146.09–3061.86), chronic cough (7.62, 1.84–31.57), constipation (2.72, 1.29–5.73)
					SUI 222/1066 (20.8)		
					UUI 13/1066 (1.2)		
					MUI 16/1066 (1.5)		
Liu and Tingting [18]	March 2013–October 2013	Guangxi	20–90 years	(Face to face) Nanning UI Epidemiological questionnaire	UI 663/2883 (23.0)	ICS definitions	SUI: Age (1.53, 1.13–2.00), chronic pelvic pain (0.73, 0.58–0.93), chronic respiratory disease (5.24, 1.17–21.27), BMI (1.53, 1.37–1.75), recurrent urinary tract infection (1.97, 1.08–3.65)
					SUI 376/2883 (13.0)		
Li and Jingxuan [19]	October 2012–June 2013	Shandong	18–90 years (married)	(Face to face) Bristol Standardized Questionnaire-Female LUTS	UI 1142/2600 (43.9)	ICS definitions	SUI: parity = 1 (1.24, 1.17–1.76), parity = 2 (2.96, 1.17–2.61), parity (3.25, 1.23–1.96), menopause (2.45, 1.01–2.35), constipation (1.39, 1.26–3.32), history of pelvic surgery (1.20, 1.00–1.73), BMI ≥ 28.0 kg/m^2^ (2.07, 1.52–3.54)
					SUI 571/2600 (22.0)		
					UUI 199/2600 (7.7)		
					MUI 372/2600 (14.3)		
							UUI: Parity = 1 (1.10, 1.17–1.76), parity = 2 (2.01, 1.17–2.61), parity ≥ U (2.93, 1.23–3.32), constipation (1.52, 1.26–3.32), BM I(2.93 kg/m^2^ (2.07, 1.52–3.54); MUI: Age 50–59 (3.15, 1.20–3.07), age 60–69 (1.55, 1.01–2.46), age 70–79 (1.25, 1.14–1.88), age ≥ 80 years (1.11, 1.10–1.96), constipation (2.01, 1.26–3.32), menopause (1.11, 1.01–1.96)
Arzigul [20]	June 2010–November 2010	Xinjiang	Y: 17–78 years	(Face to face) Questionnaires (general condition, UI prevalence situation, childbirth and chronic diseases)	SUI 338/2009 (16.8)	ICS definitions	SUI: Prolonged delivery (4.72), Difficult birth (1.85), perineum tear (1.73), chronic pelvic pain (1.50), pelvic surgery (6.68), chronic cough (2.60). (No 95% CI is reported)
Liu and Bo [21]	March 2010–September 2012	Shanghai	≥ 20 years	Bristol Standardized Questionnaire-FLUTS (face to face interviews by researchers with standardized questionnaire)	UI 1266/5433 (23.3)	ICS definitions	UI: Age (2.38, 2.16–2.63), education (2.80, 2.32–3.53), residence rural (1.95,1.64–2.31), manual labor (6.90,5.66–8.47), exercise frequency < 8 times/months (0.56, 0.47–0.68), hyperlipidemia (1.99, 1.56–2.53), no nervous system disease (0.31, 0.18–0.55), diabetes (1.69,1.20–2.38), nocturia (8.20, 6.72–10.01), constipation (7.53, 6.01–9.43), eating less greasy food (0.80, 0.73–0.88), respiratory diseases (2.67, 2.19–3.27), POP (7.37, 3.68–14.76), chronic pelvic pain (2.94, 2.20–3.91), urinary tract infection (7.75, 5.06–11.89)
					SUI 761/5433 (14.0)		
					UUI 164/5433 (3.0)		
					MUI 341/5433 (6.3)		
He and Chongjun [22]		Beijing	19–58 years (nurses)	Beijing nurse Questionnaire LUTS	SUI 349/1070 (32.6)	SUI*	SUI: Occupational stress of nurses: unclear about task expectations, business stress. (No ORs and 95% CI is reported)
					UUI 225/1070 (21.0)	UUI*	
							UUI: Occupational stress of nurses: unclear about task expectations, task conflicts, work environment, tension of interpersonal relationship. (No ORs and 95% CI is reported)
Xie and Jiangling [23]	June 2011–December 2012	Chongqing	20–78 years	(Face to face) Self-designed questionnaire (refer to Bristol Standardized Questionnaire-FLUTS)	SUI 135/666 (20.3)	ICS definitions	BMI, pelvic surgery history, hypertension, diabetes, chronic cough, constipation and other chronic diseases, perimenopausal period, multiple pregnancy abortions, parity, lateral episiotomy, perineal laceration. (No ORs and 95% CI is reported)
Jiang and Yan [24]	January 2016–May 2016	Hebei	18–91 years	(Face to face) self-designed questionnaire (refer to ICIQ-LUTS)	UI 667/2408 (27.7)	ICS definitions	UI: Age 40–49 years (5.76, 1.68–19.69), 50–59 years (8.76, 2.58–29.74), 60–69 years (8.85, 2.61–29.99), 70–79 years (6.43, 1.86–22.25), ≥ 80 years ( 4.55, 1.24–16.71), daily drinking water ≥ 1500 ml (0.75, 0.62–0.91), urinary system diseases (1.96, 1.32–2.91), respiratory diseases (1.62, 1.19–2.21), vaginitis (2.32, 1.32–4.10), abortion 1–2 times (1.51, 1.21–1.87), abortion ≥ 3 times (2.59, 1.41–4.75), hypertension (1.90, 1.52–2.38), chronic low back pain (1.52, 1.24–1.86), cesarean delivery (0.365, 0.20–0.69), postpartum infection (2.00, 1.10–3.65), dysmenorrhea (1.28, 1.04–1.57)
					SUI 557/2408 (23.1)		
					UUI 38/2408 (1.6)		
					MUI 72/2408 (3.0)		
Song and Yingchun [25]	July 2012–November 2012	Shanxi	20–87 years	(Face to face) ICIQ-LUTS	UI 882/3017 (29.2)	ICS definitions	UI: Age (1.70, 1.35–2.14), BMI (2.19, 1.68–2.84), organ prolapse (3.14, 2.49–3.95), cesarean delivery (2.38, 1.26–4.48), manual labor (2.67, 1.95–3.65), chronic cough (3.01, 1.47–6.61), constipation (2.23, 1.22–4.07), smoking (2.00, 1.37–2.91)
					SUI 547/3017 (18.1)		
					UUI 138/3017 (4.6)		
					MUI 184/3017 (6.1)		
Wang and Lihong [26]	March 2012–September 2012	GANSU	30–70 years	General survey	SUI 975/6000 (16.3)	ICS definitions	SUI: Age, parity, mode of delivery, place of residence, weight. (No ORs and 95% CI is reported)
Wan and Xiaohui [27]	April 2011to August 2011	Xinjiang	20–85 years (have sex or married)	(Face to face) ICIQ-LUTS	SUI 960/3403 (28.2)	ICS definitions	SUI: BMI (1.67, 1.08–2.58), parity (5.09, 3.89–6.67), weight of infant (5.62, 3.33–9.48), mode of delivery (2.25, 1.63–3.09), perineum lateral incision (4.45, 3.11–6.36), menopause (5.15, 3.61–7.33), chronic pelvic pain (3.87, 1.05–14.25), POP (3.50, 2.51–4.89)
					UUI 71/3403 (2.1)		
					MUI 392/3403 (11.5)		
Liu and Zhaochun [28]	July 2015–September 2015	Xinjiang	30–70 years (Gynecology and obstetrics outpatient)	(Face to face)	SUI 53/166 (31.9)	ICS definitions	SUI: Menopause, parity, delivery, puerperal incontinence. (No ORs and 95% CI is reported)
				ICIQ-UI			
Wu and Yonghong [29]	February 2013–June 2013	Guangdong	40–55 years (Perimenopausal period)	(Face to face)	SUI 211/1200 (17.6)	ICS definitions	Reported that UI was not associated with age
				ICIQ-UI			
Horng and Shiow-Shiun [30]	2005	Taiwan	35–64 years	The short form 36 (SF-36) health survey (interview survey)	UI 1036/4661 (22.2)	ICS definitions	UI: Age 44–54 (1.53, 1.27–1.83), age 55–64 (1.88, 1.49–2.37), parity 1 time (1.87, 1.17–2.99), parity 2 time (2.58, 1.73–3.85), parity ≥ 3 times (2.83, 1.90–4.23), BMI 24–26.9 kg/m^2^ (1.48, 1.25–1.76), BMI.47.0 kg/m^2^ (2.33, 1.71–3.61), history of hormone replacement therapy (1.53, 1.17–1.99), psychiatric disease (1.45, 1.07–2.07), hyperlipidemia (1.35, 1.11–1.65), respiratory disease (1.62, 1.07–2.46)
Chen and Cong [32]	January 2014–May 2014	Zhejiang	≥ 20 years	Face to face questionnaire administration, Gynecological examination	SUI 244/986 (24.7)	ICS definitions	SUI: Age > 40 years (1.4, 1.0–2.0), vaginal delivery (2.6, 1.6–4.2), hypertension (1.7, 1.1–2.5), chronic coughing (3.6, 2.1–6.2), BMI ≥ 25 kg/m^2^ (1.7, 1.1–2.4), educational level ≤ middle school (1.4, 1.4–1.9)
					UUI 47/986 (4.8)		
					MUI 53/986 (5.4)		
Hu and Mengyan [33]	May 2010–July 2010	Zhejiang	20–82 years (gynecological outpatient and married)	face to face interview, Gynecologic examination	SUI 194/500 (38.8)	SUI**	SUI: Age, BMI, vaginal delivery, hypertension, diabetes, recurrent urinary tract infection, constipation, chronic diseases, anterior and posterior vaginal wall touch, uterine prolapse. (No ORs and 95% CI is reported)
Han and Daihua [34]	January 2015–September 2016	Sichuan	35–64 years	(Face to face) ICIQ-UI, Gynecological examination	UI 455/5217 (8.7)	ICS definitions	UI: Age (0.82, 0.70–0.95), education level (1.49, 1.23–1.90), chronic bronchitis or asthma (1.55, 1.02–2.35), parity (0.79, 0.66–0.95), postpartum incontinence (8.87, 6.57–11.99), BMI (0.96, 0.93–0.99), POP (1.64, 1.09–2.49)
					SUI 348/5217 (6.7)		
					UUI 45/5217 (0.9)		
					MUI 62/5217 (1.2)		
Sun and Yanling [35]	September 2018–December 2018	Jiangsu	≥ 18 years (urban and rural)	(Face to face) ICIQ-LUTS	Urban	ICS definitions	Urban
					UI 245/443(55.3)		UI: Age (1.35, 1.04–1.43), occupation (1.82, 1.16–2.89), alcohol consumption (7.37, 1.06–28.76), smoking (1.23, 1.15–2.67), sitting > 2 h/day (5.43, 1.10–21.82)
					SUI 128/443 (28.9)		
					UUI 79/443 (17.8)		
					MUI 38/443 (8.6)		
							Rural
					Rural		UI: Age (1.06, 1.02–1.09), occupation (2.86, 1.25–6.56), alcohol consumption (7.85, 1.57–29.24), smoking (1.09, 1.01–2.52), sitting > 2 h/day (6.31, 1.59–26.86), load frequently > 3 kg (3.65, 1.38–16.79)
					UI 268/384 (69.8)		
					SUI 169/384 (44.0)		
					UUI 51/384 (13.3)		
					MUI 48/384 (12.5)		
Deng and Li [36]	July 2012–December 2012	Guangxi	22–78 years	(Face to face) ICIQ-LUTS	UI 91/1052 (37.2)	ICS definitions	UI: Age 40–49 years (1.96, 1.02–3.78), natural vaginal delivery (8.66, 4.78–15.68), perineum lateral incision (2.89, 1.64–5.27), cesarean delivery (2.31, 1.29–4.17), hypertension (1.98, 0.91–3.37), menopause (2.11, 1.10–3.56), pelvic surgery (2.38, 1.04–5.47), chronic cough (2.65, 1.43–4.92), constipation (2.11, 1.39–3.22)
					SUI 202/1052 (19.2)		
					UU I 24/1052 (2.3)		
					MUI 165/1052 (15.7)		
Wen and Fang [37]	October 2014–November 2014	Guizhou	18–70 years (married)	(Face to face) ICIQ-UI-short form	UI 68/294 (23.1)	Not reported	UI: Age (1.04, 1.01–1.07), ethnicity (2.53, 1.31–4.89), place of delivery (3.15, 1.42–6.99), mode of delivery (2.34, 1.09–5.05)
Zhang and Lijuan [38]	September 2011–June 2013	Jiangsu	20–72 years (gynecological outpatient)	Questionnaire investigation, routine physical examination	SUI 302/2655 (11.4)	Not reported	SUI: Parity (1.52, 1.13–2.36), age (1.29, 1.03–1.63), constipation (1.79, 1.30–6.84), vaginal delivery (2.95, 1.10–7.56), Prolonged delivery (3.54, 1.43–9.37), hysterectomy (3.75, 3.19–9.58), perineum laceration (1.35, 1.09–1.35)
Li and Jianjun [39]	March 2011–June 2011	Hunan	17–75 years (gynecological outpatient)	ICIQ-LUTS (long form)	SUI 122/769 (15.9)	Not reported	Not reported
					UUI 35/769 (4.6)		
					MUI 23/769 (3.0)		
Li and Fang [40]	October 2012	Shanxi	≥ 20 years (gynecological outpatient)	(Face to face) Questionnaire administration	UI 210/421 (49.9)	Not reported	UI: Age, BMI, education, daily water intake, more pregnancy, Parity, vaginal delivery, and intensity of labor. (No ORs and 95% CI is reported)
Wang and Xin [41]	January 2012–October 2012	Jilin	60–87 years (urban)	ICIQ-UI	UI 305/986 (30.9)	Not reported	UI: Respiratory diseases (1.50, 1.10–2.50), urinary diseases (2.60, 1.30–4.20), cardiovascular diseases (2.20, 1.20–4.20), gynecologic diseases (3.30, 1.90–5.40), parity > 4 times (1.80, 1.20–2.10), restriction of autonomic activity (2.40, 1.50–4.10)
					SUI 98/986 (10.0)		
					UUI 69/986 (7.0)		
					MUI 138/986 (14.0)		
Shi and Lihua [42]	April 2014–April 2015	Jiangxi	20–80 years	Bristol Standardized Questionnaire-FLUTS, pad testing	UI 150/500 (30.0)	SUI:ICS	Not reported
					SUI 93/500 (18.6)	UUI**	
					UUI 37/500 (7.4)	MUI:SUI and UUI exist at the same time	
					MUI 20/500 (4.0)		
Xin and Chunyan [43]	April 2013–September 2014	Xinjiang	≥ 65 years	(Face to face) Questionnaire administration (refer to ICIQ-UI)	UI 682/1148 (59.4)	SUI:ICS definitions	Not reported
					SUI 335/1148 (29.2)		
					UUI 83/1148 (7.2)	UUI**	
					MUI 264/1148 (23.0)	MUI*	
Zhou and Fang [44]	October 2017–December 2017	Jiangsu	18–26 years (college students)	(Face to face) ICIQ-FLUTS	UI 219/929 (23.6)	ICS definitions	Age: 21–26 years old comparing with 18–20 years old (0.87, 0.77–0.98), constipation (2.40, 1.49–3.84), alcohol consumption (1.76, 1.11–2.79), often/always delayed urination (1.74, 1.31–2.31), often/always strained to urinate (1.43, 1.11–1.85)
Chu and Lei [45]	January 2012–September 2012	Shanghai	20–83 years	(Face to face interviews) Bristol Standardized Questionnaire-FLUTS	UI 2103/7314 (28.8)	ICS definitions	UI: Postmenopausal (1.10, 1.04–1.17), pelvic surgery (1.38, 1.29–1.49), more than twice vaginal delivery history (1.49, 1.25–1.44), more than 20 weeks of pregnancy history (1.34, 1.25–1.44), the first vaginal delivery at < 20 years old (1.58, 1.46–1.71), obstetric forceps vagina delivery (2.75, 2.54–2.97), diabetes (1.16, 1.06–1.28), education (0.95, 0.90–0.99), hypertension (1.33, 1.23–1.43), BMI ≥ 30 kg/m^2^ (2.37, 2.22–2.54)
					SUI 1719/7314 (23.5)		
					UUI 154/7314 (2.1)		
					MUI 183/7314 (2.5)		
Gao and Jixue [46]	May 2010–April 2013	Shanxi	21–72 years	(Face to face) Questionnaire administration, provocative test	UI 210/589 (35.7)	ICS definitions	SUI: Age, BMI, alcohol consumption, educational level. (No ORs and 95% CI is reported)
					SUI 128/589 (21.7)		
Luo and Xiaomei [47]	March 2015–September 2015	Xizang	≥ 20 years	(Face to face) Self-designed questionnaire (health characteristics and UI occurrence)	SUI 342/1200 (28.5)	ICS definitions	SUI: Urinary tract infection (1.67, 1.42–1.98), menopause (1.22, 1.16–1.29), uterine prolapse (1.49, 1.34–1.66), Anterior vaginal prolapse (1.68, 1.63–1.73), Posterior vaginal prolapse (1.08, 1.07–1.10), BMI (1.13, 1.11–1.16), perineal laceration, postpartum labor, chronic bronchitis (1.26, 1.23–1.29), cardiovascular disease (1.16, 1.15–1.18), constipation (1.13, 1.12–1.15), occupation (1.39, 1.36–1.42), education level low (1.10, 1.09–1.11), smoking (1.13, 1.11–1.56)
Xu and Ling [48]	June 2011	Shanghai	≥ 30 years	Face to face interviews Bristol Standardized Questionnaire-FLUTS	UI 218/597 (36.5)	ICS definitions	Not reported
					SUI 104/597 (17.4)		
					UUI 32/597 (5.4)		
					MUI 82/597 (13.7)		
Wang and Yuliang [49]	October 2012–Jane 2013	Northwest, northeast, north, central, and south China	≥ 18 years	ICIQ-FLUTS (face to face interviews with questionnaires)	SUI 265/1472 (17.4)	ICS definitions	All LUTS: Age, alcohol consumption, smoking, parity (No ORs and 95% CI is reported)
					UUI 56/1472 (3.8)		
					MUI 47/1472 (3.2)		
Huang and Dong [50]	2011	Guangdong	≥ 20 years	Face to face questionnaire administration (health characteristics and UI occurrence)	UI 2373/6870 (34.5)	ICS definitions	UI: Age (1.94, 1.51–3.99), mode of delivery (2.01, 1.08–5.02), process of childbirth (2.22, 0.52–9.89), constipation (2.78, 1.77–5.52), urinary tract infection (1.31, 1.01–3.66), and oral contraceptive drugs (1.59, 1.18–3.82)
					SUI 1150/6870 (16.7)		
					UUI 309/6870 (4.5)		
					MUI 914/6870 (13.3)		
Li and Hong [51]	May 2014–March 2015	HUBEI	45–55 years (Perimenopausal period)	(Face to face) ICIQ-UI	SUI 526/2057 (25.6)	ICS definitions	SUI: Age (1.63, 1.41–1.83), parity (2.35, 1.89–2.86), POP (2.10, 1.65–4.02), pelvic surgery (2.06, 1.90–3.32), urinary tract infection (2.40, 1.76–3.05)
Lu and Shi [52]	April 2014–October 2014	Hubei	40–65 years	(Face to face) ICIQ-UI short form	UI 397/1067 (37.2)	ICS definitions	UI: Menstrual disorder (1.54, 1.06–2.24), menopause (1.41, 1.03–1.93), BMI 18.5–25.0 kg/m^2^ (2.98, 1.17–7.60), BMI > 25 kg/m^2^ (3.37, 1.24–9.12), atrophic vaginitis (1.49, 1.08–2.04), constipation (1.82, 1.32–2.51), POP (5.07, 3.37–7.63), chronic pelvic pain (1.82, 1.32–2.51), fecal incontinence (2.89, 2.31–3.34)
					SUI 344/1067 (32.2)		
					UUI 230/1067 (21.6)		
					MUI 177/1067 (16.6)		
Li and Tao [53]	April 2014–Oct ober 2014	HUBEI	40–65 years (perimenopausal)	(Face to face) ICIQ-UI-short form	SUI 504/1519 (33.2)	ICS definitions	SUI: Age: 60–65 years old (3.40, 1.92–6.04), Cesarean Sect. (0.62, 0.40–0.92), atrophic vaginitis (1.36, 1.03–1.80), constipation (1.44, 1.07–1.93), chronic pelvic pain (2.17, 1.90–4.03), POP (2.81, 1.36–5.79), fecal incontinence (3.32, 2.03–5.43), monthly income: 2000–3999 comparing with ≤ 1999 (0.063, 0.40–0.92)
					UUI 366/1519 (24.1)		
					MUI 264/1519 (17.4)		
Lu and Shi [54]	April 2014–December 2014	HUBEI	40–65 years (perimenopausal)	(Face to face) ICIQ-UI-short form	UI 585/1519 (38.5)	ICS definitions	UI: Age: over 50 years old (2.0, 1.21–3.29), overweight (BMI: 25–30 kg/m^2^) (2.85, 1.33–6.07), irregular menstruation (1.45, 1.03–2.06), gynecological diseases (1.31, 1.01–1.70), chronic pelvic pain (2.32, 1.48–3.64), POP (2.33, 1.61–3.37), constipation (1.42, 1.07–1.88), fecal incontinence (3.29, 2.33–4.65)
					SUI 482/1519 (31.9)		
					UUI 366/1519 (24.1)		
					MUI 264/1519 (17.4)		
Wang and Bingyi [55]	No time is reported	Beijing	35–64 years	(Face to face) ICIQ-UI-short form	UI 59/126 (46.8)	ICS definitions	Age (No ORs and 95% CI is reported)
Chang and kengming [56]		Taiwan	≥ 60 years	Face to face interview	UI 485/1517 (32.0)	ICS definitions	UI: Age (1.04, 1.02–1.07), diabetes mellitus (1.65, 1.11–2.47), previous urinary disease (3.46, 2.26–5.30), BMI (1.06, 1.01–1.11)
Li and Ruixia [57]	Apr 2013–Jun 2013	Shanghai	60–70 yeas	(Face to face) ICIQ-UI	UI 332/1962 (16.9)	ICS definitions	UI: BMI, pregnancy, pregnancy leakage, gynecological diseases, constipation, urinary tract infection, chronic respiratory disease. (No ORs and 95% CI is reported)
					SUI 237/1962 (12.1)		
Gao and Maolong [58]	January 2013–May 2013	Beijing	≥ 60 years	(Face to face) Author-designed Questionnaire (Social and health characteristics)	UI 164/694 (23.6)	Patient reported	UI: Age 80–84 years (1.72, 0.96–3.08), age ≥ 85 years (2.49, 1.22–5.06), malnutrition (1.69, 1.08–2.65), chronic pain (1.47, 1.01–2.14), cognitive disorder (1.56, 1.08–2.24), sleep disorders (1.76, 1.22–2.53), drugs 1–4 types (2.23, 1.04–4.76), drug ≥ 5 types (5.07, 2.30–11.19)
Li and Yan [59]	2017	Sichuan	60–97 years (pension institutions)	(Face to face) ICIQ-UI-short form,	UI 102/205 (49.8)	ICS definitions	UI: BMI < 24 kg/m^2^ (0.38, 0.18–0.78), spouse (2.54, 1.20–5.38), Mental labour (0.25, 0.12–0.53), no alcohol consumption (0.40, 0.16–0.96), no delayed voiding (0.12, 0.05–0.30), number of chronic disease < 2 types (0.39, 0.18–0.83), parity ≥ 4 (2.82, 1.18–6.75), age of the first parity < 21 years (2.76, 1.03–7.43)
					SUI 35/205 (17.1)		
					UUI 21/205 (10.2)		
					MUI 41/205 (20.0)		
Chen and Mina [60]	No time is reported	Zhejianng	60–100 years (medical and nursing institutions)	(Face to face) ICIQ-UI-short form,	UI 217/352 (61.6)	ICS definitions	Education (0.59, 0.41–0.86), income (0.58, 0.37–0.92), medical payment method (1.64, 1.09–2.49), have no other disease (0.27, 0.13–0.58), activities of daily living (1.37, 1.12–1.67), indwelling catheterization (13.31, 2.35–75.22)
Zhu and Zhichao [61]	January 2014–December 2014	Zhejiang	20–81 years	ICIQ-UI	UI 301/1178 (25.5)	UI*	UI: Age (1.02, 1.00–1.05), natural vaginal delivery (2.53, 1.10–5.84), difficult vaginal delivery (4.54, 1.63–12.64), Cesarean delivery (3.07, 1.10–1.45), parity (1.10, 1.00–1.21), BMI 20–25 kg/m^2^ (1.43, 1.14–1.80), BMI > 25 kg/m^2^ (1.40, 1.17–1.89), hypertension (1.32, 1.01–1.74), diabetes (1.49, 1.17–1.89), other diseases (1.21, 1.02–1.45)
Che and Xinyan [62]	July 2018	Beijing	Mean: 39.4 ± 9.9 years (medical staff)	(Face to face) ICIQ-UI-short form,	SUI 63/146(43.2)	ICS definitions	Constipation (4.95, 1.81–13.53), Natural delivery (3.50, 1.49–8.21)
Liu and Yanjuan [63]	Nov 2010–Nov 2011	Hebei	≥ 20 years (railway workers)	(Face to face) ICIQ-UI	UI 474/1368 (34.6)	ICS definitions	Not reported
					SUI 296/1368 (21.6)		
					UUI 52/1368 (3.8)		
					MUI 126/1368 (9.2)		
Li and Hui [64]	2014	Liaoning	≥ 18 years	(Face to face) ICIQ-LUTS	UI 1063/3456 (30.8)	ICS definitions	Not reported
					SUI 750/3456 (21.7)		
					UUI 97/3456 (2.8)		
					MUI 176/3456 (5.1)		

*E* English-language articles searched from PubMed. *POP* pelvic organ prolapse

^a^All the questionnaires used in the studies were pencil and paper questionnaires

^b^ICIQ-FLUTS: Chinese version of the International Consultation on Incontinence Questionnaire-Female Lower Urinary Tract Symptoms

^c^ICS: International Continence Society

*UI**: [88]

SUI*: Urine leakage during exercise

SUI**: In the past year, loss of urine more than twice when abdominal pressure is increased (including stress, laughing, coughing, etc.)

UUI*: Urinating without any warning or a small amount of early warning, sudden stimulation results in uncontrolled urine outflow

UUI**: Occurrence of urinary frequency, urgency, increased frequency of nocturnal discharge and decreased urine output, or cannot control, too late, leading to urine leakage

MUI*: SUI and UUI exist at the same time

## Abbreviations

BMI: Body Mass Index
ICS: International Continence Society
MUI: Mixed urinary incontinence
POP: Pelvic organ prolapse
SUI: Stress urinary incontinence
UI: Urinary incontinence
UUI: Urgency urinary incontinence

## References

[1] Haylen BT, de Ridder D, Freeman RM, Swift SE, Berghmans B, Lee J (2010). An International Urogynecological Association (IUGA)/International Continence Society (ICS) joint report on the terminology for female pelvic floor dysfunction. Neurourol Urodyn.

[2] Aoki Y, Brown HW, Brubaker L, Cornu JN, Daly JO, Cartwright R (2017). Urinary incontinence in women. Nat Rev Disease Primers.

[3] Searcy JAR (2017). Geriatric urinary incontinence. Nurs Clin North Am.

[4] Juliato CR, Baccaro LF, Pedro AO, Gabiatti JR, Lui-Filho JF, Costa-Paiva L (2017). Factors associated with urinary incontinence in middle-aged women: a population-based household survey. Int Urogynecol J.

[5] Schreiber Pedersen L, Lose G, Hoybye MT, Elsner S, Waldmann A, Rudnicki M (2017). Prevalence of urinary incontinence among women and analysis of potential risk factors in Germany and Denmark. Acta Obstet Gynecol Scand.

[6] Marit HE, Steinar H, Guri R, Yngvild SH (2013). Prevalence, incidence and remission of urinary incontinence in women: longitudinal data from the Norwegian HUNT study (EPINCONT). BMC Urol.

[7] Wu JM, Hundley AF, Fulton RG (2009). Forecasting the prevalence of pelvic floor disorders in US women 2010–2050. Obst Gynecol.

[8] Zhang L, Zhu L, Xu T, Lang J, Li Z, Gong J (2015). A population-based survey of the prevalence, potential risk factors, and symptom-specific bother of lower urinary tract symptoms in adult Chinese Women. Eur Urol.

[9] Zhou F, Newman DK, Palmer MH (2018). Urinary urgency in working women: What factors are associated with urinary urgency progression?. J Women's Health.

[10] Coyne KS, Kvasz M, Ireland AM, Milsom I, Kopp ZS, Chapple CR (2012). Urinary incontinence and its relationship to mental health and health-related quality of life in men and women in Sweden, the United Kingdom, and the United States. Eur Urol.

[11] Hung KJ, Awtrey CS, Tsai AC (2014). Urinary incontinence, depression, and economic outcomes in a cohort of women between the ages of 54 and 65 years. Obstet Gynecol.

[12] Wilson L, Brown JS, Shin GP, Luc KO, Subak LL (2001). Annual direct cost of urinary incontinence. Obstet Gynecol.

[13] Chang KM, Hsieh CH, Chiang HS, Lee TS (2017). Trends in inpatient female urinary incontinence surgery and costs in Taiwan, 1997–2011. Taiwan J Obstet Gynecol.

[14] Milsom I, Coyne KS, Nicholson S, Kvasz M, Chen CI, Wein AJ (2014). Global prevalence and economic burden of urgency urinary incontinence: a systematic review. Eur Urol.

[15] Palmer MH, Wu JM, Marquez CS, Rupp B, Conover MM, Newman DK (2019). "A secret club": focus groups about women's toileting behaviors. BMC Womens Health.

[16] The State Council of Office of Population Censuses and Surveys. Data of the 2010 Census in China. China Statistics Press. 2012, 3.

[17] Sun WY (2014). The analysis of high risk factors on the adult female urinary incontinence of Qinghai.

[18] Liu TT (2014). Nanning city related risk factors of female stress urinary incontinence and biomimetric electrical stimulation treatment.

[19] Li JX (2014). Epidemiologic investigation of married woman with urinary incontinence in rural areas in Laiwu city.

[20] Arzigul A (2013). Epidemiology and risk factors of female stress urinary incontinence in some counties and townships of Xinjiang of Hotan.

[21] Liu B, Wang L, Huang SS (2014). Prevalence and risk factors of urinary incontinence among Chinese women in Shanghai. Int J Clin Exp Med.

[22] He CJ, Zhang CF, Hai T, Yu LP, Wang Q, Gu BW (2013). Prevalence of overactive bladder and other lower urinary tract symptoms in female nurses in Beijing and its association with occupational stress. Chin J Urol.

[23] Xie JL, She X, Yang YW, Chen D (2013). A survey on the incidence and risk factors of adult female stress urinary incontinence in Southeast Chongqing. J Mod Urol.

[24] Jiang Y, Yan L, Du FD, Zhen PT, Zhang L, Jiang L (2016). Prevalence and associated factors of female urinary incontinence in Hebei province. Chin J Obstet Gynecol.

[25] Song YC, Zhang PL, Xie L, Wang SQ, Zhao XL, Fan LJ (2014). Study on the epidemiology of urinary incontinence and the impact on the quality of life of adult female in Xi'an. Shanxi Med J.

[26] Wang LH (2014). Analysis of 1830 cases with female pelvic floor dysfunction in Suzhou district of Jiuquan City Gansu Province. Chin J Family Plan Gynecotokol.

[27] Wan XH, Ding Y, Gulina A, Zainuer A, Lin L, Manrepa T (2013). Epidemiologic study on the risk factors of the adult female urinary incontinence in Uygur of Kashi in Xinjiang. Chin J Obstet Gynecol.

[28] Liu ZC, Wei XH, Liu XL, Luo MM, Hao Y (2015). Investigation on female stress urinary incontinence in reclamation area. China Modern Doctor.

[29] Wu YH, Hu HW, Han CY (2014). Survey on the current status of stress urinary incontinence among women during their menopausal transition in communities of Nanshan District. Shenzhen Chin Gener Pract.

[30] Horng S-S, Huang N, Wu S-I, Fang Y-T, Chou Y-J, Chou P (2013). The epidemiology of urinary incontinence and it's influence on quality of life in Taiwanese middle-aged women. Neurourol Urodyn.

[31] Chang KM, Hsieh CH, Chiang HS, Lee TS (2014). Risk factors for urinary incontinence among women aged 60 or over with hypertension in Taiwan. Taiwan J Obst Gynecol.

[32] Chen C, Lu Y, Peng JW, Hu YJ, Lin XH, Wu XQ (2016). Epidemiologic investigation on pelvic floor dysfunction among female in Wenzhou. J Wenzhou Med Univ.

[33] Hu MY (2013). Investigation and analysis of related factors of stress urinary incontinence of married women in Jiaojiang. Chin J Rural Med.

[34] Han DH, Yuan JY, Bin XY, Yan LP (2017). Prevalence and influence factors of urinary incontinence in 35–64 year old women in Chengdu Shuangliu District. J Pub Health Prev Med.

[35] Sun Y, Xue K, Su M, Xu H, Zhou F (2019). Investigation and analysis of urinary incontinence in urban and rural adult women in Xuzhou City, Jiangsu Province. J Nurs Train.

[36] Deng L, Wei YP, Zhang LY, Liu J, Liu LL, Huang Y (2013). Epidemiological survey of adult female urinary incontinence in Nanning. J Guangxi Med Univ.

[37] Wen F, Yang YJ, Zi D, Luo C, Chen GQ, Wu JJ (2017). The status and comparison of 294 women with pelvic floor dysfunction dieseases among Miao, Buyi and Han nationalities. J Guizhou Med Univ.

[38] Zhang LJ, Chen YF, Qin WH, Huang HP (2015). Epidemiological investigation on pelvic floor dysfunction in Wuxi. China Matern Child Health Care.

[39] Li JJ, Guo HC, Wang XL, Wang BN, Wei J (2013). Investigation and analysis of urinary tract symptoms among Changsha hospital gynecological clinic women. Guangdong Med J.

[40] Li F, Liu J, Lie TT (2014). An investigation of risk factors for adult female stress urinary incontinence in outpatient clinic. Shanxi Med J.

[41] Wang X, Li ZG, Wang W, Hou J, Li JQ (2013). Prevalence, quality of life and risk factors of urinary incontinence of elderly women in Jilin City. Chin J Gerontol.

[42] Shi LH, Wang WH, Li P, Tang HY, Zhang FY, Cao H (2016). A survey of the prevalence of stress urinary incontinence of women in Pingxiang. Contemp Med.

[43] Xin CY, Ge YH, Zhang CP (2014). Incidence of urinary incontinence and quality of life of women aged 65 years and older in four communities in Urumqi. Chin J Gerontol.

[44] Zhou F, Xue K, Liu Y, Zhuo L, Tu S, Palmer MH (2019). Toileting behaviors and factors associated with urinary incontinence in college-aged female students in China. Int Urogynecol J.

[45] Chu L, Wang JJ, Fan BZ, Sun J, Cao GY, Xu MJ (2015). The prevalence and risk factors of urinary incontinence among women in Shanghai. Prog Obstet Gynecol.

[46] Gao JX, He XL, Li Y, Bai AS, Guo W, Ma YD (2015). Epidemiological analysis of female stress urinary incontinence in Yan'an City. J Kunming Med Univ.

[47] Luo XM, Zhuoma, Liu CH, Ren NJ, Li D, Chen W (2017). Epidemiological investigation of female stress urinary incontinence in Shannan City. J Clin Res.

[48] Xu L, Yang Y (2013). Status quo of adult female urinary incontinence and quality of life in Shanghai. J Shanghai Jiaotong Univ (Med Sci).

[49] Wang Y, Hu H, Xu K, Wang X, Na Y, Kang X (2015). Prevalence, risk factors and the bother of lower urinary tract symptoms in China: a population-based survey. Int Urogynecol J.

[50] Huang D, Yang MY, Lin XM, Zhang L (2013). Epidemiological studies of adult female urinary incontinence in Zhanjiang. China Matern Child Health Care.

[51] Li H, Liu XL, Cheng L (2015). Epidemiological investigation and analysis of factors affecting stress urinary incontinence in climacteric women. China Matern Child Health Care.

[52] Lu S, Zhang H-L, Zhang Y-J, Shao Q-C (2016). Prevalence and risk factors of urinary incontinence among perimenopausal women in Wuhan. J Huazhong Univ Sci Technol (Med Sci).

[53] Li T, Zhang YJ, Zhang HL, Ding XH, Yu ZJ, Lu S (2019). Prevalence and risk factors of stress urinary incontinence among perimenopausal women and its influence on daily life in women with sexual desire problem. Curr Med Sci.

[54] Lu S, Zhang Y, Ding X, Zhang H (2017). Investigation on the influencing factors of urinary incontinence among perimenopausal women in some areas of Hubei Province. Mater Child Health Care China.

[55] Wang B, Zhang A (2019). The incidence of urinary incontinence among women aged 35 to 64 in Changping district of Beijing and its impact on quality of life. Womens Health Res.

[56] Chang KM, Hsieh CH, Chiang HS, Lee TS (2014). Risk factors for urinary incontinence among women aged 60 or over with hypertension in Taiwan. Taiwan J Obstet Gynecol.

[57] Li RX, Ma M, Xiao XR, Xu Y, Li B (2015). Analysis of prevalence and life quality and associared factors of stress urinary incontinence of the women aged 60–70 years in Shanghai. Geriatr Health Care.

[58] Gao ML, Wang J, Wang JT, Song YT (2014). Study on the prevalence of urinary incontinence among elderly people of residential community in Beijing city and the risk factors. Pract Geriatr.

[59] Li Y, Zhang X, Liang X, Wang H, Zheng Y (2019). Analysis and intervention of risk factors of urinary incontinence in elderly care institutions. Chin J Gerontol.

[60] Chen M, Chen X, Ding W, Zhang T, Feng W, Wang Q (2018). Survey of prevalence and influencing factors of elderly patients with urinary incontinence in medical and nursing institutions. Nurs Res.

[61] Zhu ZC, Zhu JM, Shao SH, Jiang RJ (2016). Epidemiological investigation of female urinary incontinence in Yuhang City. Chin J Hum Sex.

[62] Che X, Wu S, Chen Y, Huang Y, Yang Y (2019). A survey of risk factors and quality of life in female medical staff with urinary incontinence. J Peking Univ (Health Sci).

[63] Liu YJ, Qiao J, Yan H (2013). Prevalence of urinary incontinence and stress urinary incontinence on quality of life among female workers in Baoding railway Hebei Province. Occup Health.

[64] Li H, Bai F, Zhang J, Bai H, Sun B (2014). Epidemiological investigation of urinary incontinence in rural women in Liaoning. Chin For Health Digest.

[65] Harlow BL, Bavendam TG, Palmer MH, Brubaker L, Burgio KL, Lukacz ES (2018). The Prevention of Lower Urinary Tract Symptoms (PLUS) research consortium: a transdisciplinary approach toward promoting bladder health and preventing lower urinary tract symptoms in women across the life course. J Womens Health.

[66] Zhang N, He Y, Wang J, Zhang Y, Ding J, Hua KQ (2016). Effects of a new community-based reproductive health intervention on knowledge of and attitudes and behaviors toward stress urinary incontinence among young women in Shanghai: a cluster-randomized controlled trial. Int Urogynecol J.

[67] Pelaez M, Gonzalez-Cerron S, Montejo R, Barakat R (2014). Pelvic floor muscle training included in a pregnancy exercise program is effective in primary prevention of urinary incontinence: a randomized controlled trial. Neurourol Urodyn.

[68] Paul Abrams, Wagg A. Incontinence. 6th ed. 2017.

[69] Zhu L, Li L, Lang J-H, Xu T (2012). Prevalence and risk factors for peri- and postpartum urinary incontinence in primiparous women in China: a prospective longitudinal study. Int Urogynecol J.

[70] Palmer MH, Cockerell R, Griebling TL, Rantell A, van Houten P, Newman DK. Review of the 6th International Consultation on Incontinence: Primary prevention of urinary incontinence. Neurourol Urodyn. 2019. 10.1002/nau.24222 31737950

[71] Hagglund D, Olsson H, Leppert J (1999). Urinary incontinence: an unexpected large problem among young females. Results from a population-based study. Fam Pract.

[72] O'Halloran T, Bell RJ, Robinson PJ, Davis SR (2012). Urinary incontinence in young nulligravid women: a cross-sectional analysis. Ann Intern Med.

[73] Mishra GD, Barker MS, Herber-Gast GC, Hillard T (2015). Depression and the incidence of urinary incontinence symptoms among young women: results from a prospective cohort study. Maturitas.

[74] Bardino M, Di Martino M, Ricci E, Parazzini F (2015). Frequency and determinants of urinary incontinence in adolescent and young nulliparous women. J Pediatr Adolesc Gynecol.

[75] Da Roza T, Brandao S, Mascarenhas T, Jorge RN, Duarte JA (2015). Urinary incontinence and levels of regular physical exercise in young women. Int J Sports Med.

[76] Casey EK, Temme K (2017). Pelvic floor muscle function and urinary incontinence in the female athlete. Phys Sportsmed.

[77] Palmer MH, Willis-Gray MG, Zhou F, Newman DK, Wu JM (2017). Self-reported toileting behaviors in employed women: Are they associated with lower urinary tract symptoms?. Neurourol Urodyn.

[78] Wan X, Wu C, Xu D, Huang L, Wang K (2017). Toileting behaviours and lower urinary tract symptoms among female nurses: a cross-sectional questionnaire survey. Int J Nurs Stud.

[79] Palmer MH, Newman DK (2015). Women's toileting behaviours: an online survey of female advanced practice providers. Int J Clin Pract.

[80] Liu Y (2013). Prevalence of lower urinary tract symptoms in female nurses and its relationship with toileting behavior.

[81] Zhu L, Lang J, Wang H, Han S, Huang J (2008). The prevalence of and potential risk factors for female urinary incontinence in Beijing. China Menopause.

[82] Walters MD (2015). Urinary incontinence in women comes and goes, and reasons remain elusive. BJOG.

[83] Zhang L (2015). A population-based epidemiology survey of the lower urinary tract symptoms in adult Chinese women—cross-sectional study.

[84] Volløyhaug I, Mørkved S, Salvesen Ø, Salvesen K (2015). Pelvic organ prolapse and incontinence 15–23 years after first delivery: a cross-sectional study. BJOG.

[85] Reigota RB, Pedro AO, de Souza Santos Machado V, Costa-Paiva L, Pinto-Neto AM (2016). Prevalence of urinary incontinence and its association with multimorbidity in women aged 50 years or older: a population-based study. Neurourol Urodyn.

[86] Kasikci M, Kilic D, Avsar G, Sirin M (2015). Prevalence of urinary incontinence in older Turkish women, risk factors, and effect on activities of daily living. Arch Gerontol Geriatr.

[87] Erekson EA, Cong X, Townsend MK, Ciarleglio MM (2016). Ten-year prevalence and incidence of urinary incontinence in older women: a longitudinal analysis of the health and retirement study. J Am Geriatr Soc.

[88] Jian F, Liyan Z (2003). Urinary incontinence classification criteria and stress urinary incontinence diagnosis. J Pract Obst Gynecol.

